# Prevalence of Treatment Resistant Psychoses in a Complete National Forensic Mental Health Service: A Dundrum Forensic Redevelopment Evaluation Study (D-FOREST)

**DOI:** 10.1192/j.eurpsy.2023.928

**Published:** 2023-07-19

**Authors:** M. U. Waqar, H. Amin, E. Ní Mhuircheartaigh, H. G. Kennedy, M. Davoren

**Affiliations:** 1 The Dundrum Centre for Forensic Excellence, Trinity College Dublin; 2 National Forensic Mental Health Service, Dublin, Ireland

## Abstract

**Introduction:**

Treatment resistant schizophrenia and other treatment resistant psychotic disorders are believed to be over-represented in forensic patient clusters. The true rates of treatment resistant psychoses in secure forensic hospitals remain unexplored.

**Objectives:**

This study aimed to ascertain the prevalence of treatment resistant psychoses within a complete national forensic mental health service. In addition, the study sought to examine the relationships between treatment resistance for psychotic symptoms and treatment resistance in other domains, such as offending behaviour.

**Methods:**

This is a cross-sectional study of a complete cohort of patients admitted to the National Forensic Mental Health Service in Ireland during the period 01/11/2021 to 31/01/2022. All inpatients at the time of the study were included. Demographic details, data appertaining to diagnosis, medication, measures of risk (HCR-20), recovery (DUNDRUM toolkit), functioning (GAF), and symptoms (PANSS) were collated. Data were gathered as part of the Dundrum Forensic Redevelopment Evaluation Study (D-FOREST).

**Results:**

The sample consisted of 170 patients. Majority (n=162) 95.3% were male. The majority (n=116), 68.2%, were admitted from prisons, while a smaller number (n=35), 20.6%, were admitted from other psychiatric facilities. The insanity defense (n=94) 55.3% was the most common legal status, followed by unfit to plead (n=16) 9.4%. The commonest diagnosis was schizophrenia (n=97) 57.1%, followed by schizoaffective disorder (n=27) 15.9% and autism spectrum disorder (n=5) 2.9%. The mean age at admission was 35.52 years and the median age was 34.37 ± 9.43 SD.

Of the total sample, 25.9% of patients were on more than 1000 mg per day chlorpromazine equivalent (CPZE) doses. Those whose psychotic symptoms required treatment with CPZE doses over 1000 mg per day scored poorly on DUNDRUM-3 programme completion, DUNDRUM-4 recovery scale, HCR-20 historical, HCR-20 clinical, HCR-20 risk, HCR-20 dynamic, and had poorer overall functioning (all *P*<0.001) than those who required lower antipsychotic doses. On binary logistic regression, correcting for age and gender, the only variable that remained significant was GAF (adjusted odds ratio = 0.979, 95% CI 0.962-0.996, *P*=0.014). In forward entry model regression, only the DUNDRUM-4 recovery scale (odds ratio = 1.13, 95% CI 1.07-1.19, *P*<0.001) and GAF (adjusted odds ratio = 0.979, 95% CI 0.962-0.996, *P*<0.001) were significant. This model had a robust forward and backward likelihood ratio.

**Conclusions:**

Rates of treatment resistant psychoses in forensic hospital groups are indeed elevated. Overall functioning on GAF and recovery across a wide range of components in the DUNDRUM-4 scale are the best predictors of treatment resistant psychosis.

**Disclosure of Interest:**

None Declared

